# Adherence to cancer screening guidelines across Canadian provinces: an observational study

**DOI:** 10.1186/1471-2407-10-304

**Published:** 2010-06-18

**Authors:** Erin C Strumpf, Zhijin Chai, Srikanth Kadiyala

**Affiliations:** 1 Department of Epidemiology, Biostatistics and Occupational Health, McGill University Leacock 418, 855 Sherbrooke St. West, Montreal, QC H3A 2T7, Canada; 2 Department of Economics, McGill University Leacock 418, 855 Sherbrooke St. West, Montreal, QC H3A 2T7, Canada; 3 McGill University, c/o Erin Strumpf, 855 Sherbrooke St. West, Montreal, QC H3A 2T7, Canada; 4 Department of Pharmacy, University of Washington 1959 NE Pacific St., Seattle, WA 9819, USA

## Abstract

**Background:**

Cancer screening guidelines reflect the costs and benefits of population-based screening based on evidence from clinical trials. While most of the existing literature on compliance with cancer screening guidelines only measures raw screening rates in the target age groups, we used a novel approach to estimate degree of guideline compliance across Canadian provinces for breast, colorectal and prostate cancer screening. Measuring compliance as the change in age-specific screening rates at the guideline-recommended initiation age (50), we generally found screening patterns across Canadian provinces that were not consistent with guideline compliance.

**Methods:**

We calculated age-cancer-specific screening rates for ages 40-60 using the Canadian Community Health Survey (2003 and 2005), a cross-sectional, nationally representative survey of health status, health care utilization and health determinants in the Canadian population. We estimated the degree of compliance using logistic regression to measure the change in adjusted screening rates at the guideline-recommended initiation age for each province in the sample.

**Results:**

For breast cancer, after adjusting for age trends and other covariates, being above age 50 in Quebec increased the probability of being screened by 19 percentage points, from an average screening rate of 24% among 40-49 year olds. None of the other regions exhibited a statistically significant change in screening rates at age 50. Additional analyses indicated that these patterns reflect asymptomatic screening and that Quebec's breast cancer screening program enhanced the degree of guideline compliance in that province. Colorectal cancer screening practice was consistent with guidelines only in Saskatchewan, as screening rates increased at age 50 by 12 percentage points, from an average rate of 6% among 40-49 year olds. For prostate cancer, the regions examined here are not compliant with Canadian guidelines since screening rates were quite high, and there was not a discrete increase at any particular age.

**Conclusions:**

Screening practice for breast, colorectal and prostate cancer was generally not consistent with Canadian clinical guidelines. Quebec (breast) and Saskatchewan (colorectal) were exceptions to this, and the impact of Quebec's breast cancer screening program suggests a role for policy in improving screening guideline compliance.

## Background

Clinical guidelines codify and transmit existing knowledge regarding best practice and aid physicians and patients in choosing optimal treatment. The extent to which Canadian patients and physicians follow, or comply with, such guidelines for cancer screening remains largely unknown. Studies that examine Canadian cancer screening practice generally conclude that breast and colorectal cancer screening rates are low, relative to the guideline recommendation that everyone in a given age group be screened, while prostate cancer screening rates are high. In 2003, 26% of women had not had a mammogram within the last two years [1] and 58% of Canadians ages 50 and over had never had a fecal occult blood test (FOBT) or endoscopy [2]. Meanwhile, approximately half of men over age 50 have received a prostate-specific antigen (PSA) test in their lifetime [3] and men between the ages of 50 to 69 are two- to three-times more likely to be screened for prostate cancer than colorectal cancer, even though PSA testing is not generally recommended in Canada [4]. These and other existing studies generally focus on screening rates in the age group recommended for screening [5-7].

By specifying the age at which screening should begin, the guidelines implicitly recommend that screening not occur for asymptomatic individuals below that age, often due to low specificity of the test and the health consequences of unnecessary intervention and treatment. A high screening rate among the target age group does not necessarily reflect compliance if, for example, screening rates are also high among age groups not recommended for screening. We therefore expect that in regions where guidelines are being followed, a sharp change in population screening rates will be evident at the guideline-recommended initiation age. We measured guideline compliance using a novel measure: the change in screening rates among age groups below the recommended initiation age and those above the threshold. Only two other studies conceive of compliance in this way [8,9], but neither examined population screening rates. We believe this is an important complimentary definition of screening compliance that more accurately reflects the guidelines themselves and has implications for both clinical practice and health care policy.

This study examined the degree of compliance with national screening guidelines for breast, colorectal, and prostate cancer, defined as the change in screening rates at the guideline-recommended initiation age. Specifically, we used logistic regression to test for a statistically significant change in adjusted screening rates at the guideline-recommended initiation age. We also investigated how this definition of compliance varies across Canadian provinces and addressed the role of provincial screening programs for breast cancer.

### Cancer screening guidelines and provincial cancer screening programs

Table 1 presents cancer screening guidelines from the Canadian Task Force on Preventive Health Care and the Canadian Cancer Society that were in place during the study period (2003-2005). These guidelines were based on results from clinical trials and specified which screening tests should be used, the periodicity of screening, and the target population [10]. These evidence-based recommendations were relevant across Canada for asymptomatic individuals without a family history of cancer.

**Table 1:** Canadian cancer screening guidelines from the Canadian Task Force on Preventive Health Care (CTFPHC) and the Canadian Cancer Society (CCS)

	Breast Cancer	Colorectal Cancer	Prostate Cancer
	**CTFPHC (2001) **[44]	**CTFPHC (2001) **[45]	**CTFPHC (2000) **[46]

Test	Mammography	FOBT (good evidence); sigmoidoscopy (fair evidence); insufficient evidence to include or exclude colonoscopy	PSA
Periodicity	Every 1-2 years	Every 1 to 2 years; periodicity unspecified	Not be used due to low positive predictive value and risk of adverse affects associated with treatment
Population	Asymptomatic women ages 50-69	Asymptomatic individuals age 50+	NA
			
	**CCS (2005) **[47]	**CCS(2005) **[48]	**CCS (2005) **[49]

Test	Mammography	FOBT	PSA
Periodicity	Every 2 years	At least every 2 years	Discuss with physician the benefits and risks
Population	Asymptomatic women ages 50-69	Asymptomatic individuals age 50+	Men age 50+

*No changes have occurred with the recommendations set forth by the Canadian Cancer Society since 1997.

Provincial screening programs also have the potential to influence screening rates and compliance with guidelines. All ten provinces had breast cancer screening programs in place during the study period, with British Columbia starting in 1988 and Prince Edward Island being the last to introduce a program in 1999. All programs focused on mammography as the preferred screening method and targeted asymptomatic women ages 50-69, except BC which screened women ages 40-79 [11-20]. These programs aimed to increase screening rates via awareness (advertising and individual reminder letters) and improved access (mobile screening units), but their impact on compliance with guidelines is unknown. Organized screening programs for colorectal and prostate cancer are not relevant given the time frame of this study, since Alberta, Manitoba and Ontario began colorectal cancer screening programs in 2007 and BC only recently initiated a program for prostate cancer screening [21-24].

## Methods

### Study population and data

We estimated age-specific screening rates using the 2003 and 2005 Canadian Community Health Survey (CCHS) microdata files accessed through a Statistics Canada Research Data Center. The CCHS is a cross-sectional, nationally representative survey of health status, health care utilization and health determinants in the Canadian population with an overall response rate of 81% in 2003 and 79% in 2005 [25,26]. The analyzed regions varied across cancer type due to differences in the administration of survey questions across provinces and minimum sample size requirements from Statistics Canada. For breast cancer, we examined screening rates in the Atlantic region (Newfoundland and Labrador, Prince Edward Island, Nova Scotia and New Brunswick combined), Quebec, Ontario, the Prairies (Manitoba, Saskatchewan and Alberta combined), and British Columbia. All health regions administered the breast cancer screening module. For colorectal cancer, we analyzed the Atlantic region, Ontario, Saskatchewan and British Columbia and for prostate cancer, the Atlantic region (not including Nova Scotia), Ontario and British Columbia. We used two years of the CCHS because the colorectal and prostate cancer screening questions were asked in different provinces in different years (colorectal cancer screening: Saskatchewan (7 of 11 health regions) and British Columbia in 2003, Atlantic provinces and Ontario in 2005; prostate cancer screening: Ontario (24 of 37 health regions) and British Columbia in 2003, Atlantic provinces in 2005. All health regions unless otherwise noted.).

We collected screening and expenditure data from provinces' breast cancer screening programs, as well as the date the program began and their target population [27-37]. Most provincial programs were already in existence over the time period for which CCHS screening data exist, and only limited expenditure data was available. We combined this information with data from the National Population Health Survey (1994/95-1998/99) and the CCHS from 2001-2005 to evaluate the impact of screening programs on guideline compliance.

### Statistical analysis

To estimate guideline compliance, separate cross-sectional logistic regressions were estimated for each type of cancer, by region, on a gender-appropriate sample of adults ages 40-60. Dependent variables indicated receipt of screening in the past two years for breast (mammography), colorectal (sigmoidoscopy, colonoscopy or FOBT), and prostate cancer (PSA test). This measure captured a "point-in-time" estimate of the percent of individuals screened at different ages and did not focus on individuals' screening histories over time. An indicator variable for being above the guideline-recommended initiation age (age 50+) was the key independent variable. Covariates included age trends (age, age^2^, age^3^), sex (for colorectal screening), race (white and non-white), education (less than secondary, secondary graduate, some post-secondary, and post-secondary graduate), household income quartile, and marital status (married, divorced or separated, widowed, never married, and unmarried couple). We converted adjusted odds-ratios to relative risks by calculating the ratio of predicted probabilities of being above versus below the age threshold, with confounders at their mean (i.e., marginal effects) [38]. All analyses accounted for the survey's complex sampling frames and data were weighted to be nationally representative.

To allay concerns that our results were driven by a sharp increase in symptomatic screening at age 50, and therefore should not be interpreted in the context of guideline compliance, we ran a parallel analysis to that described above. Here the dependent variable equaled one if the respondent's reason for receiving the test in the past two years was "age" or "regular check-up" and equal to zero if they received the test for another reason (family history, previously detected lump, breast problem, hormone replacement therapy, or follow-up treatment) or have not received the test in the past two years.

Organized provincial breast cancer screening programs are likely to increase screening rates, but whether they improve adherence to clinical guidelines is an open question. Because screening programs may affect screening rates and guideline compliance through several different avenues ranging from increased publicity and awareness to increased access to screening outside physicians' offices, we continued to use the self-reported measure of screening from any source from the CCHS. Data limitations allowed us to examine only the impact of the introduction of Quebec's program (Programme québécois de dépistage du cancer du sein) in 1998, by comparing the change in screening rates at age 50 before the program was in place to the change at age 50 after implementation. In a logistic regression similar to that outlined above, an indicator variable equal to zero in years before the program's start and one after was interacted with the variable indicating age 50 or above.

## Results

### Breast cancer

**Figure 1:**
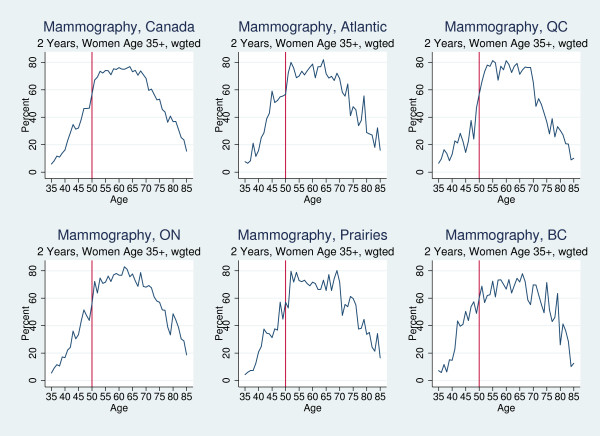
Percent of Women Who Report Mammography Screening in the Previous Two Years, by Year-of-Age, 2005.

Figure 1 shows the percent of Canadian women who had a mammogram in 2005 by region and by year of age. In Canada overall, screening rates increased significantly from 47% at age 49, to 57% and 66% at ages 50 and 51, respectively. When broken down by province, Quebec demonstrated the clearest adherence to the guidelines, with screening rates increasing from 48% at age 49 to 67% at 51. Ontario displayed a similar but less substantial jump at age 50, while the other three regions showed no apparent change. The graphs suggest that physicians and women followed the national guidelines in at least some regions. However, they also show a general upward trend in mammography rates as women age and possible discrete shifts at ages other than 50. For example, the screening rate in British Columbia almost doubled from 13% at age 39, to 25% at age 41, potentially indicating a trend break at age 40 that did not adhere to national guidelines but was consistent with BC's provincial screening program. Trend breaks at age 40 for breast cancer screening would also be consistent with compliance U.S. breast cancer screening guidelines. We used regression analysis to test whether these apparent trend breaks at different ages persisted after controlling for age trends and other confounders.

Across provinces, logistic regression analysis showed that being over age 50 increased breast cancer screening rates by 9.9 percentage points [OR = 1.49; p = .002] (Kadiyala S and Strumpf EC: Are U.S. and Canadian cancer screening rates consistent with guideline information regarding the age of screening initiation?, submitted). Table 2 presents the logistic regression results and shows that, after adjusting for age trends and other covariates, being above age 50 in Quebec increased the probability of being screened by 19.3 percentage points [OR = 2.19; p = 0.004] (Additional file 1, available in an attachment, provides the full set of regression coefficients). None of the other regions studied exhibited significant changes in screening rates at age 50. A separate regression analysis (not shown) examined the impact of being over age 40 on screening rates among women ages 35-55. Mammography rates did not change significantly at age 40 after controlling for covariates in British Columbia, but in Quebec [OR = 2.014; p = 0.049] and the Prairie provinces [OR = 2.261; p = 0.026] instead. It is worth noting that, although women in higher income quartiles were more likely to be screened in all provinces except Quebec, income was not generally a significant predictor of guideline compliance.

**Table 2:** Impact of guideline initiation age on cancer screening - adjusted odds ratio of being at or above the guideline recommended initiation age

Dependent variable breast: Received mammogram in the past 2 years Dependent variable colorectal: Received FOBT test, sigmoidoscopy or colonoscopy in the past 2 years Dependent variable prostate: Received PSA test in the past 2 years			
	**Adjusted Odds Ratio (95% Confidence Interval)**		

	**Breast**	**Colorectal**	**Prostate**

Atlantic	1.70	0.92	1.22
	(0.86, 3.35)	(0.45, 1.89)	(0.50, 2.96)

Quebec	2.19**		
	(1.29, 3.72)		

Ontario	1.32	1.12	1.16
	(0.85, 2.05)	(0.74, 1.69)	(0.63, 2.12)

Saskatchewan		3.62*	
		(1.09, 12.05)	

Prairies	1.31		
	(0.71, 2.42)		

British Columbia	1.21	0.99	1.01
	(0.60, 2.43)	(0.52, 1.89)	(0.48, 2.12)

*** p <= .001, **p <= .01, * p <= .05. Notes: Each coefficient comes from a separate logistic regression which includes controls for age, age^2 ^and age^3^; race (white/non-white), marital status (5 categories), education (4 categories), household income quartiles, and sex (for colorectal).

Marginal effects for the age cutoff variable (breast): Atlantic 12.95, Quebec 19.34, Ontario 6.87, Prairies 6.76, British Columbia 4.69

Marginal effects for the age cutoff variable (colorectal): Atlantic -0.82, Ontario 1.48, Saskatchewan 12.51, BC -0.11

Marginal effects for the age cutoff variable (prostate): Atlantic 4.37, Ontario 3.14, British Columbia 0.21

Sample sizes (breast, women ages 40-60): Atl N = 2,745, QC N = 4,885, ON N = 6,341, Prairies N = 3,849, BC N = 2,324

Sample sizes (colorectal, adults ages 40-60): Atl N = 5,005, ON N = 12,112, SK N = 1,149, BC N = 4,722

Sample sizes (prostate, men ages 40-60): Atl N = 1,533, ON N = 3,353, BC N = 2,230

Note: Additional file 1, available in an attached document, presents the full set of regression coefficients for each of these models.

### Colorectal cancer

**Figure 2:**
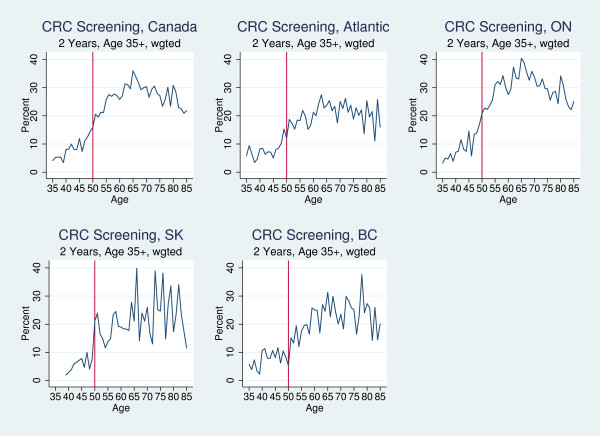
Percent of Adults Who Report Colorectal Cancer Screening in the Past Two Years, by Year-of-Age, 2003 and 2005.

Despite clear clinical guidelines and the inclusion of several different screening methods in this analysis, colorectal cancer screening rates were quite low. Figure 2 plots colorectal screening by age for men and women combined. Screening rates rose from 15% at age 49 to 17% at age 50 and 21% at age 51 in Canada. These rates showed a more visible change collectively at the age 50 threshold than each region separately, with the exception of Saskatchewan.

Across provinces, regression results showed a 1.3 percentage point increase in colorectal cancer screening rates at age 50 [OR = 1.11; p = .53] (Kadiyala S and Strumpf EC: Are U.S. and Canadian cancer screening rates consistent with guideline information regarding the age of screening initiation?, submitted). Table 2 presents the regression results for colorectal screening by region, controlling for age trends and other covariates, including gender. Being above the age threshold in Saskatchewan increased the probability of being screened by 12.5 percentage points [OR = 3.62; p = 0.036]. In contrast, we did not observe significant changes in screening rates at age 50 in the Atlantic provinces, Ontario, or British Columbia.

### Prostate cancer

**Figure 3:**
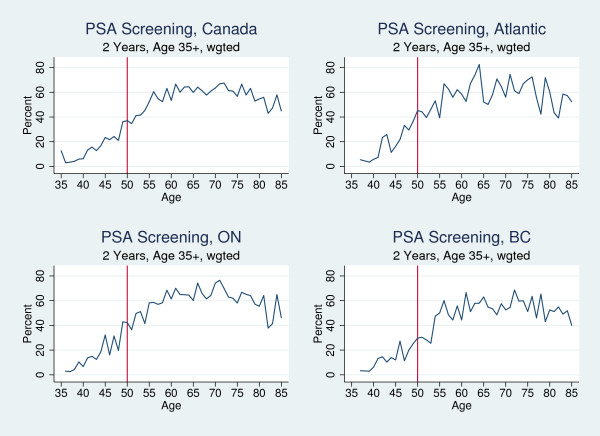
Percent of Men Who Reported Prostate Cancer Screening in the Past Two Years, by Year-of-Age, 2003 and 2005.

While national guidelines do not recommend the use of PSA tests, Figure 3 shows that age-specific rates were relatively high in Canada. About 35 percent of 50 year old men reported having received a PSA test in the past two years. However, beyond the trend of screening rates increasing with age, there did not appear to be a discrete increase at any particular age in Canada overall or in any of the regions we examined.

Across provinces, there was no significant change in prostate cancer screening rates at age 50 [OR = 1.043, p = .843] (Kadiyala S and Strumpf EC: Are U.S. and Canadian cancer screening rates consistent with guideline information regarding the age of screening initiation?, submitted). Table 2 presents the regression results by region controlling for age trends and demographics. Consistent with Figure 3, it shows no statistically significant change in adjusted screening rates at age 50.

### Preventive vs. symptomatic screening

The national cancer screening guidelines only applied to asymptomatic individuals without a family history of the disease. The results using asymptomatic screening as the dependent variable matched those presented above very closely. For breast cancer, there was a sharp increase in asymptomatic screening rates of 17 percentage points at age 50 in Quebec [OR 2.42, p = 0.002, CI 1.39-4.22], but not in any other region. For colorectal cancer, there was a very small increase in asymptomatic screening at age 50 only in Saskatchewan [OR .002, CI 0.00-0.84], suggesting that the increase shown in Table 2 is due to "symptomatic" reasons. None of the regions exhibited a statistically significant change in asymptomatic prostate cancer screening rates at age 50.

### The role of provincial screening programs

After controlling for the same set of demographic variables and adding year indicators to control for changes in screening rates over time, breast cancer screening rates for women age 50 and above were 25 percentage points higher after the initiation of the Quebec program than for the same age group before the program was in place [OR 2.81, CI 1.71, 4.61]. This result indicates that our measure of guideline compliance increased significantly after program implementation. Notably, because no organized screening programs for prostate or colorectal cancer were in place at the time of this study, the rates presented here for those screening types can be interpreted as baseline, pre-program rates.

## Discussion

This study examined the degree of compliance with cancer screening guidelines across Canadian provinces, defined as the change in screening rates at the guideline-recommended initiation age. We found that after controlling for covariates, breast cancer screening rates in Quebec increased at age 50 by 19 percentage points and in Saskatchewan, colorectal cancer screening rates increased at age 50 by nearly 13 percentage points. No other region demonstrated compliance with screening guidelines for these cancers and no region demonstrated compliance with prostate cancer screening guidelines. For breast cancer, these patterns reflected screening among asymptomatic individuals.

Previous research in this area has focused on measuring screening rates and identifying physician- or patient-level predictors of screening. Beyond describing "high" or "low" cancer screening rates, we defined a novel and complementary definition of screening guideline compliance and identified variation in compliance across regions. The main result was that breast, colorectal and prostate cancer screening rates do not change sharply at the guideline-recommended initiation age, suggesting fairly low compliance rates among the provinces and regions examined here. Breast cancer screening practice in Quebec was quite different, and the analysis of the impact of the provincial screening program suggested that organized screening programs can affect not only overall screening rates, but guideline compliance as well. Of course the Quebec case may not be generalizable, but more extensive data are required to conduct similar analyses in other provinces. Colorectal cancer screening rates were quite low, and with the exception of Saskatchewan, the regions studied here did not comply with the guideline-recommended initiation age. On the other hand, prostate cancer screening rates were higher than the guidelines recommend, but we also failed to see a sharp change in clinical practice at any particular age. We interpreted this as lack of compliance with Canadian screening guidelines. Future research may address whether recent findings regarding the effectiveness of PSA testing and changing U.S. clinical guidelines affect Canadian compliance rates [39-41].

While Canadian guidelines recommended that breast and colorectal cancer screening begin at age 50, the realities of clinical practice mean that screening begins "around" that age. We looked for sharp changes in screening rates at age 50 in Figures 1, 2 and 3, but our interpretation of the regression analysis only required that average screening rates among 50-60 year olds were significantly higher than among 40-49 year olds. If respondents reported having been screened at 52 or 53, this was consistent with the guidelines and our interpretation of the results. Furthermore, the 2-year screening window allowed us to capture screening at age 48 or 49 as being guideline compliant as well.

### Limitations

Due to the self-reported nature of screening in the CCHS, it is possible that the screening rates reported here were subject to recall bias. However, as long as any bias with respect to accuracy or recency does not change sharply at age 50, we do not expect such a bias to affect the change in screening rates at the guideline-recommended initiation age. In fact, analysis of screening received in the last year yields results similar to those presented here for a 2-year timeframe. If, however, respondents age 50 and above are differentially accurate in their self-reports relative to younger respondents, self-reported data could overstate the degree of guideline compliance. Existing evidence suggests that the sensitivity of reported screening is very high across age categories, but it is also sparse, suggesting the need for additional research on this question to help validate this measure of guideline compliance [42].

A second limitation stems from the fact that not all provinces included questions on all types of cancer screening in the CCHS. Unless this was due to reasons correlated with cancer screening patterns or policy, as opposed to the length and cost of surveys, the results presented here are unlikely to be biased. However, this study has demonstrated that screening patterns for different cancers differ greatly across provinces, and therefore inferences should not be drawn about provinces not represented in the data. Lastly, these data did not allow us to distinguish whether guideline compliance operated through patient behavior, physician behavior, or both.

Several other limitations stem from our study design and variable definitions. First, we focused on screening in the last two years, but the 5-10 year periodicity recommended for sigmoidoscopy, colonoscopy and double-contrast barium enemas by organizations other than the CTFPHC and the CCS [43] mean that we may have understated the degree of colorectal cancer screening guideline compliance over a longer window. Furthermore, because we were interested in the change in screening rates at the guideline initiation age, we focused on a narrow range of ages (40-60) where we believed that other determinants of screening were relatively similar. This means, however, that we did not include individuals who were screened above age 60 as contributing to "compliance", although by more standard definitions they do. Third, we characterized our estimates in terms of a percentage change in screening rates at age 50 relative to the average rate below this age. As a result, a large absolute change on a high base rate yielded a smaller percentage change than a smaller absolute change on a lower base rate. We believe this is of greater importance across cancers than across provinces, but presented both raw and percentage changes so that the reader can interpret the data in either context.

## Conclusions

This analysis presented a novel definition of compliance with cancer screening guidelines. We found low compliance with cancer screening guidelines across Canadian provinces and identified an opportunity to improve compliance by reducing screening rates among younger groups and increasing them among the guideline-recommended age group. Improved compliance could have implications for both the efficacy and cost-effectiveness of population-based cancer screening in Canada as well as other countries.

Several avenues exist for further research in this area, most notably examining the relationship between guideline compliance and cancer-related morbidity and mortality. Identifying patient- and provider-level factors that predict compliance and examining the impact of provincial colorectal cancer screening programs are other important areas for future research.

## Competing interests

The authors declare that they have no competing interests.

## Authors' contributions

ECS contributed substantially to conception and design, acquisition of data, analysis and interpretation of data, drafted the article, and revised it critically for important intellectual content. ZC contributed substantially to acquisition of data, analysis of data, drafted portions of the article, and revised it critically for important intellectual content. SK contributed substantially to conception and design and revised the article critically for important intellectual content. All authors read and approved the final manuscript.

## Pre-publication history

The pre-publication history for this paper can be accessed here:

http://www.biomedcentral.com/1471-2407/10/304/prepub

## Supplementary Material

Additional file 1 **Impact of guideline initiation age on cancer screening: full model Expanded version of Table **2, **will all the regression coefficients included in the models**. Click here for file
