# Supplementary material for: Adherence to cancer screening guidelines across Canadian provinces: an observational study
Source: BMC Cancer. 2010 Jun 18;10:304. doi: 10.1186/1471-2407-10-304 (PMC2908097; doi:10.1186/1471-2407-10-304)
Supplement: Additional file 1 — Impact of guideline initiation age on cancer screening: full model Expanded version of Table 2, will all the regression coefficients included in the models. [file 1471-2407-10-304-S1.DOC]

**Erin Strumpf, “Adherence to cancer screening guidelines across Canadian provinces: an observational study”**

**Additional file 1: Impact of guideline initiation age on cancer screening: full model**

Impact of guideline initiation age on breast cancer screening

| Women ages 40-60  Dependent variable: Received mammogram in the past 2 years | | | | | |
| --- | --- | --- | --- | --- | --- |
|  | Adjusted Odds Ratio  (95% Confidence Interval) | | | | |
|  | Atlantic | Quebec | Ontario | Prairies | British Columbia |
| Age>=50 | 1.70 | 2.19** | 1.32 | 1.31 | 1.21 |
|  | (0.86, 3.35) | (1.29, 3.72) | (0.85, 2.05) | (0.71, 2.42) | (0.60, 2.43) |
| Race non-white | 2.18* | 1.10 | 1.12 | 0.94 | 0.90 |
|  | (1.10, 4.33) | (0.67, 1.82) | (0.85, 1.50) | (0.63, 1.34) | (0.63, 1.29) |
| Income quartile 2 | 1.33 | 1.13 | 1.15 | 1.09 | 1.20 |
|  | (0.94, 1.88) | (0.78, 1.64) | (0.80, 1.66) | (0.69, 1.71) | (0.77, 1.87) |
| Income quartile 3 | 2.10*** | 1.01 | 1.37 | 1.29 | 1.42 |
|  | (1.47, 3.02) | (0.72, 1.40) | (0.99, 1.88) | (0.86, 1.94) | (0.95, 2.13) |
| Income quartile 4 | 2.28*** | 1.05 | 1.36 | 1.69 | 1.70* |
|  | (1.49, 3.50) | (0.72, 1.53) | (0.98, 1.90) | (1.08, 2.64) | (1.11, 2.62) |
| Common law marriage | 0.68 | 0.99 | 0.81 | 0.84 | 0.80 |
|  | (0.43, 1.08) | (0.74, 1.32) | (0.60, 1.09) | (0.54, 1.31) | (0.54, 1.21) |
| Widowed | 0.66 | 0.55* | 0.52** | 1.12 | 0.67 |
|  | (0.41, 1.09) | (0.31, 0.97) | (0.33, 0.81) | (0.59, 2.13) | (0.40, 1.13) |
| Single | 0.84 | 1.07 | 0.75 | 0.71 | 0.88 |
|  | (0.55, 1.29) | (0.78, 1.46) | (0.56, 1.00) | (0.49, 1.04) | (0.61, 1.27) |
| Divorced/ separated | 1.08 | 0.88 | 0.98 | 0.67 | 0.80 |
|  | (0.74, 1.57) | (0.64, 1.21) | (0.76, 1.28) | (0.46, 0.98) | (0.54, 1.18) |
| Secondary graduate | 1.53* | 1.26 | 1.20 | 1.17 | 2.25** |
|  | (1.05, 2.22) | (0.87, 1.82) | (0.87, 1.65) | (0.73, 1.87) | (1.35, 3.75) |
| Some post-secondary | 1.21 | 1.42 | 1.36 | 1.26 | 1.50 |
|  | (0.69, 2.14) | (0.89, 2.26) | (0.85, 2.17) | (0.73, 2.17) | (0.86, 2.60) |
| Post-secondary graduate | 1.25 | 1.10 | 1.49** | 1.49 | 1.39 |
|  | (0.91, 1.70) | (0.82, 1.49) | (1.11, 2.01) | (0.99, 2.25) | (0.90, 2.17) |
|  |  |  |  |  |  |
| N | 2,745 | 4,885 | 6,341 | 3,849 | 2,324 |
| *** p<=.001, **p<=.01, * p<=.05. Notes: Logistic regression includes controls for age, age^2^ and age^3^; income quartiles based on household income. | | | | | |
| Marginal effects for the age cutoff variable: Atlantic 12.95, Quebec 19.34, Ontario 6.87, Prairies 6.76, British Columbia 4.69 | | | | | |

**Additional file 1 (cont.): Impact of guideline initiation age on cancer screening: full model**

Impact of guideline initiation age on colorectal cancer screening

| Adults ages 40-60  Dependent variable: Received FOBT test, sigmoidoscopy or colonoscopy in the past 2 years | | | | |
| --- | --- | --- | --- | --- |
|  | Adjusted Odds Ratio  (95% Confidence Interval) | | | |
|  | Atlantic | Ontario | Saskatchewan | British Columbia |
| Age>=50 | 0.92 | 1.12 | 3.62* | 0.99 |
|  | (0.45, 1.89) | (0.74, 1.69) | (1.09, 12.05) | (0.52, 1.89) |
| Race non-white | 0.93 | 0.96 | 0.92 | 0.61* |
|  | (0.52, 1.68) | (0.76, 1.22) | (0.29, 2.92) | (0.41, 0.91) |
| Income quartile 2 | 0.98 | 1.03 | 1.02 | 0.89 |
|  | (0.71, 1.35) | (0.74, 1.43) | (0.45, 2.34) | (0.56, 1.41) |
| Income quartile 3 | 1.02 | 0.90 | 1.13 | 0.90 |
|  | (0.74, 1.39) | (0.68, 1.19) | (0.53, 2.38) | (0.60, 1.34) |
| Income quartile 4 | 1.05 | 1.13 | 0.83 | 1.04 |
|  | (0.74, 1.50) | (0.86, 1.49) | (0.37, 1.86) | (0.70, 1.57) |
| Common law marriage | 0.74 | 1.09 | 0.30 | 1.17 |
|  | (.47, 1.19) | (0.84, 1.43) | (0.08, 1.20) | (0.65, 2.10) |
| Widowed | 0.50* | 0.83 | 1.69 | 1.28 |
|  | (0.27, 0.95) | (0.53, 1.29) | (0.60, 4.79) | (0.64, 2.56) |
| Single | 0.72 | 0.98 | 0.47 | 0.81 |
|  | (0.51, 1.01) | (0.77, 1.23) | (0.21, 1.07) | (0.55, 1.20) |
| Divorced/ separated | 0.71* | 1.14 | 0.78 | 0.87 |
|  | (0.51, 0.99) | (0.89, 1.47) | (0.37, 1.65) | (0.63, 1.20) |
| Secondary graduate | 0.76 | 1.12 | 1.52 | 0.92 |
|  | (0.52, 1.11) | (0.85, 1.49) | (0.79, 2.94) | (0.56, 1.52) |
| Some post-secondary | 0.63 | 1.03 | 0.51 | 0.64 |
|  | (0.35, 1.16) | (0.74, 1.45) | (0.17, 1.51) | (0.34, 1.19) |
| Post-secondary graduate | 0.93 | 1.38** | 0.99 | 0.82 |
|  | (0.69, 1.24) | (1.08, 1.75) | (0.53, 1.85) | (0.53, 1.29) |
| Female | 0.90  (0.71, 1.14) | 0.92  (0.80, 1.07) | 0.88  (0.54, 1.41) | 1.04  (0.80, 1.36) |
|  |  |  |  |  |
| N | 5,005 | 12,112 | 1,149 | 4,722 |
| *** p<=.001, **p<=.01, * p<=.05. Notes: Logistic regression includes controls for age, age^2^ and age^3^; income quartiles based on household income. | | | | |
| Marginal effects for the age cutoff variable: Atlantic -0.82, Ontario 1.48, Saskatchewan 12.51, BC -0.11 | | | | |

**Additional file 1 (cont.): Impact of guideline initiation age on cancer screening: full model**

Impact of guideline initiation age on prostate cancer screening

| Men ages 40-60  Dependent variable: Received PSA test in the past 2 years | | | |
| --- | --- | --- | --- |
|  | Adjusted Odds Ratio  (95% Confidence Interval) | | |
|  | Atlantic | Ontario | British Columbia |
| Age>=50 | 1.22 | 1.16 | 1.01 |
|  | (0.50, 2.96) | (0.63, 2.12) | (0.48, 2.12) |
| Race non-white | 1.90 | 0.89 | 0.60* |
|  | (0.88, 4.09) | (0.61, 1.32) | (0.38, 0.94) |
| Income quartile 2 | 1.09 | 0.64 | 0.94 |
|  | (0.64, 1.86) | (0.39, 1.06) | (0.46, 1.93) |
| Income quartile 3 | 1.87* | 1.07 | 0.95 |
|  | (1.13, 3.11) | (0.68, 1.69) | (0.48, 1.89) |
| Income quartile 4 | 1.66 | 1.31 | 1.14 |
|  | (0.97, 2.85) | (0.83, 2.08) | (0.56, 2.33) |
| Common law marriage | 0.71 | 0.67 | 0.79 |
|  | (0.38, 1.33) | (0.44, 1.02) | (0.46, 1.34) |
| Widowed | 2.39 | 0.77 | 0.38 |
|  | (0.80, 7.14) | (0.30, 1.92) | (0.10, 1.44) |
| Single | 0.52* | 0.82 | 0.68 |
|  | (0.30, 0.91) | (0.53, 1.28) | (0.39, 1.19) |
| Divorced/ separated | 0.56* | 0.58** | 0.84 |
|  | (0.36, 0.89) | (0.41, 0.82) | (0.52, 1.36) |
| Secondary graduate | 1.19 | 1.36 | 1.80* |
|  | (0.70, 2.04) | (0.89, 2.07) | (1.01, 3.22) |
| Some post-secondary | 1.10 | 1.51 | 1.41 |
|  | (0.45, 2.70) | (0.91, 2.51) | (0.72, 2.77) |
| Post-secondary graduate | 1.18 | 1.51* | 1.67 |
|  | (0.77, 1.80) | (1.06, 2.17) | (0.98, 2.84) |
|  |  |  |  |
| N | 1,533 | 3,353 | 2,230 |
| *** p<=.001, **p<=.01, * p<=.05. Notes: Logistic regression includes controls for age, age^2^ and age^3^; income quartiles based on household income. | | | |
| Marginal effects for the age cutoff variable: Atlantic 4.37, Ontario 3.14, British Columbia 0.21 | | | |
